# Profil et qualité de vie de patients atteints de polyarthrite rhumatoïde en Guinée Conakry et au Cameroun

**DOI:** 10.11604/pamj.2021.38.379.20098

**Published:** 2021-04-19

**Authors:** Aly Badra Kamissoko, Paul Eloundou, Marie Traoré, Mamadou Lamine Diallo, Gervais Mendo, M’balou Fatoumata Diallo, Alhassane Diallo

**Affiliations:** 1 Service de Rhumatologie, Hôpital National Ignace Deen, Conakry, Guinée,; 2 Service de Médecine Interne Hôpital de District d´Efoulan, Yaoundé, Cameroun,; 3 Service de Rhumatologie, Hôpital Sud Francilien, Corbeil Essonne, France,; 4 Department of Epidemiology, Biostatistics, and Clinical Research, Assistance Publique-Hôpitaux de Paris, Hospital Bichat, University Paris Diderot, Paris, France

**Keywords:** Polyarthrite rhumatoïde, qualité de vie, activité de la maladie, Afrique noire, Rheumatoid arthritis, quality of life, disease activity, Black Africa

## Abstract

**Introduction:**

la polyarthrite rhumatoïde (PR) altère de façon considérable la qualité de vie des patients. L´objectif de notre étude était d´étudier le lien entre l´activité de la maladie et la qualité de vie de patients guinéens et camerounais atteints de polyarthrite rhumatoïde.

**Méthodes:**

étude pilote transversale multicentrique (Hôpital national Ignace Dean de Conakry en Guinée et Hôpital de district d´Efoulan Yaoundé au Cameroun) de 15 mois (1^er^ octobre 2016 au 30 janvier 2018). Le diagnostic de PR était basé sur les critères de l´ACR/EULAR (American college of rheumatology, European League against Rheumatism). Le questionnaire EMIR (échelle de mesure de l'impact de la polyarthrite rhumatoïde) et le score de Steinbrocker ont été utilisés pour évaluer la qualité de vie.

**Résultats:**

cinquante-deux patients dont 82% de femmes ont été colligés. Le score EMIR total était de 5,06±0,50 considéré comme une qualité de vie relativement altérée. L´altération de la qualité de vie était plus marquée sur les composants psychiques (6,78±0,99) et douleur (5,37±0,99). Le composant travail était le moins affecté (4,03±0,98). Le DAS28 était significativement lié aux composants psychique (p=0,036 ; R=0,29), douleur (p=0,076 ; R=0,25), physique (p=0,0029; R=0,41) et à la qualité de vie globale (EMIR total) (p=0,027 ; R=0,31).

**Conclusion:**

l´impact le plus significatif de la PR sur la qualité de vie est corrélé à la douleur (EVA-douleur) et à l´activité de la maladie (DAS 28). Les résultats de cette étude pilote devront être confirmés par une étude d´une plus grande ampleur.

## Abstract (en)

**Introduction:**

rheumatoid arthritis (RA) dramatically affects the quality of life of patients. The objective of our study was to study the link between the activity of the disease and the quality of life of Guinean (Conakry) and Cameroonian patients with RA.

**Methods:**

pilot multicentric cross-sectional study (Ignace Dean National Hospital of Conakry in Guinea and Efoulan Yaoundé District Hospital in Cameroon) for 15 months (1^st^ October 2016 to 30^th^ January 2018). The diagnosis of RA was based on the criteria of the ACR/EULAR. Disease activity was assessed by DAS 28. The EMIR questionnaire and the Steinbrocker score were used to assess quality of life.

**Results:**

fifty-two patients, 82% of whom were women. The total EMIR score was 5.06±0.50 as a relatively impaired quality of life. Alteration of quality of life was more marked on psychic components (6.78±0.99) and pain (5.37±0.99). The work component was the least affected (4.03±0.98). DAS28 was significantly related to psychic components (p=0.036, R=0.29), pain (p=0.076, R=0.25), physical (p=0.0029, R=0.41), and at the overall quality of life (total EMIR) (p=0.027, R=0.31).

**Conclusion:**

the most significant of RA on quality of life was related to pain (EVA-pain) and RA activity (DAS 28). The results of this pilot study will have to be confirmed by a largest study.

## Introduction

La polyarthrite rhumatoïde (PR) est le rhumatisme inflammatoire chronique le plus fréquent [1]. En Guinée (Conakry), la prévalence hospitalière de la PR est de 0,09 % à l´hôpital national Ignace Deen [2]. Au Cameroun, cette prévalence hospitalière est de 0,04% à l´hôpital de district d´Efoulan [3]. La PR reste encore méconnu par le personnel médical en Afrique subsaharienne ce qui se traduit par d´importants retards diagnostiques [4]. Cette pathologie altère de façon considérable la qualité de vie du patient qui en est atteint [5]. Il existe des échelles, présentées sous la forme d'auto-questionnaire, qui complètent utilement l'évaluation médicale [6,7]. Des études africaines ont évalué la qualité de vie des patients atteints de PR [6-9]. En Afrique subsaharienne, la qualité de vie a été sur les aspects socio-économiques [9] et la répercussion des complications de la maladie [10,11]. A notre connaissance, le lien entre l´activité de la maladie et la qualité de vie de la PR dans cette région n´a pas été établi. Nous nous sommes proposé d´étudier chez des patients guinéens et camerounais la qualité de vie (sur plusieurs aspects) ainsi que son lien avec l´activité de la polyarthrite rhumatoïde.

## Méthodes

**Patients, type d´étude et variables**: nous avons réalisé une étude transversale multicentrique de 15 mois (du 1^er^ octobre 2016 au 30 janvier 2018) sur des patients atteints de PR dans deux centres, le service de rhumatologie de l´Hôpital national Ignace Deen de Conakry (Guinée) et le service de médecine interne de l´Hôpital de district d´Efoulan Yaoundé (Cameroun). Étaient inclus dans l´étude, les patients âgés de plus de 18 ans et atteints de PR diagnostiqués selon les critères ACR/EULAR 2010 [12] et consultant dans les deux sites d´étude. Nous n´avons pas inclus les patients ayant un antécédent psychiatrique et ceux ayant une autre maladie altérant leur condition physique. Les patients étaient ou pas sous traitement de fond de la PR. Le consentement était requis après explication de la procédure et de l´objectif de l´étude. Les variables suivantes ont été recueillies: 1) variables sociodémographiques: âge, sexe, situation financière, niveau scolaire. 2) variables clinico-biologiques: indice de masse corporelle, périmètre abdominal, échelle visuelle analogique, nombre d´articulations douloureuses, nombre d´articulations gonflées, nombre de réveils nocturnes, dérouillage matinal, délai diagnostique, facteur rhumatoïde, anticorps anti-peptides cycliques citrullinés. Le DAS 28 (Disease Activity Score) a été classé ainsi: PR en rémission si le DAS 28 < 2,6, PR de faible activité si le DAS 28< 3,2 ; PR modérée si le DAS 28 varie entre 3,2 et 5,1 inclus; PR de forte activité DAS 28 > 5,1. 3) le retentissement de la maladie: le reclassement professionnel, le score de Steinbrocker [13] et l´échelle EMIR (échelle de mesure de l´impact de la polyarthrite rhumatoïde) [14]. L´échelle EMIR court comporte cinq composants et 26 questions (réponses concernant les quatre dernières semaines). A chaque composant correspond un nombre de questions donné: EMIR physique (1 à 12), EMIR douleur (13 à 15), EMIR psychique (16 à 20), EMIR social (20 à 24), EMIR travail (25 et 26) [14].

**Analyse des données**: les variables qualitatives ont été résumées par la fréquence et le pourcentage, et les variables quantitatives par la moyenne et l´écart-type. Les comparaisons entre l´EMIR et DAS28 d´une part, et entre l´EMIR et l´EVA-douleur d´autre part ont été effectuées par un test de corrélation de Pearson (ou Spearman). Pour identifier les facteurs de risque associés à l´âge de début des symptômes, nous avons utilisé un modèle de régression linéaire. Les variables associées à l´âge de début des symptômes avec une p-value < 0,10 en univarié ont été entrées dans le modèle multivarié. Ensuite, les facteurs de risque indépendants de l´âge de début des symptômes ont été sélectionnés par une procédure pas à pas descendante. Tous les tests étaient bilatéraux, le seuil de significativité a été fixé à 5%, et toutes les analyses ont été faites par le logiciel R.

**Considérations éthiques**: le consentement éclairé des patients a été obtenu et le protocole accepté par le comité d´éthique de l´Hôpital national Ignace Deen et de l´Hôpital de district d´Efoulan Yaoundé.

## Résultats

Nous avons colligé 52 cas de PR (42 en Guinée et 10 au Cameroun) dont 43 femmes (82,69%). L´âge moyen des patients au début de la maladie était de 36,25±20,44 ans (Tableau 1). Deux-tiers des patients (35 cas soit 67,31%) étaient financièrement dépendants d´une tierce personne (Tableau 1). La majorité avait une PR d´activité forte (27 patients soit 51,92%) (Tableau 1). L´évaluation de la capacité fonctionnelle a montré un stade III de Steinbrocker (autorisant le sujet à effectuer seulement une petite partie de ses occupations usuelles et de ses propres soins) chez 33 patients (63,46%) (Tableau 1). Nous avons comparé les caractéristiques cliniques et la qualité de vie entre les patients Guinéens (Conakry) et Camerounais (Tableau 1 et Tableau 2). A l´exception de l´activité de la maladie à travers le DAS 28 (p=0,0015) et du dérouillage matinal (p<0,0001), nous n´avons pas mis en évidence de différence entre ces deux populations. Alors qu´en moyenne l´activité de la maladie était plus forte chez les patients Guinéens (DAS 28: 5,69±1,19 vs 4,48±0,89; p=0,0015), le dérouillage matinal était plus long chez les patients Camerounais. Nous avons recherché les facteurs associés à la capacité fonctionnelle selon la classification de Steinbrocker.

**Tableau 1:** caractéristiques de la polyarthrite rhumatoïde chez les patients guinéens et camerounais

Variables	Guinée(n=42)	Cameroun(n=10)	Global (n=52)
Age (années)	46,81±15,66	48,40±12,46	47,12±15,00
Âge de début de la maladie (années)	43,10±15,85	40,90±11,20	42,67±14,99
Sexe (féminin)	34(80,95%)	9(90%)	43(82,69%)
Situation financière (dépendante)	30(71,43%)	5(50%)	35(67,31%)
Délai diagnostique (années)	51,74±52,04	43,10±98,83	50,08±62,55
IMC (kg/cm^2^)	24,15±5,79	27,46±8,68	24,78±6,48
Périmètre abdominal(cm)	88,88±22,53	90,30±21,81	89,15±22,19
EVA	58,93± 21,11	68±7,89	60,67&plumn;19,55
NAD	12±6	6±2	11±6
NAG	3±2	5±1	3±2
Réveil nocturne	3±1	3±1	3±1
Dérouillage matinal	1,55±0,67	3,00±1,05	1,83±0,94
FR(positif)	17(50%)	6(67%)	23(58,97)
Anti-CCP(positif)	6(60%)	3(42,85%)	9(52,94%)
DAS 28	5,69±1,19	4,48±0,82	5,44±1,22
Rémission	1(2,38%)	1(10%)	2(3,84%)
Faible activité	1(2,38%)	5(50%)	6(11,53%)
Activité modérée	3(7,14%)	4(40%)	7(13,46%)
Forte activité	27(64,28%)	0(0%)	27(51,92%)

**EVA:** échelle visuelle analogique; **NAD:** nombre d´articulations douloureuses; **NAG:** nombre d’articulations gonflées; **FR:** facteur rhumatoïde; **anti-CCP:** anticorps anti-peptide cyclique citrulliné; **DAS:** disease activity score. Les données sont représentées par la moyenne (± écart-type) pour les variables quantitatives et l´effectif (fréquence) pour les variables qualitatives.

**Figure 1:**
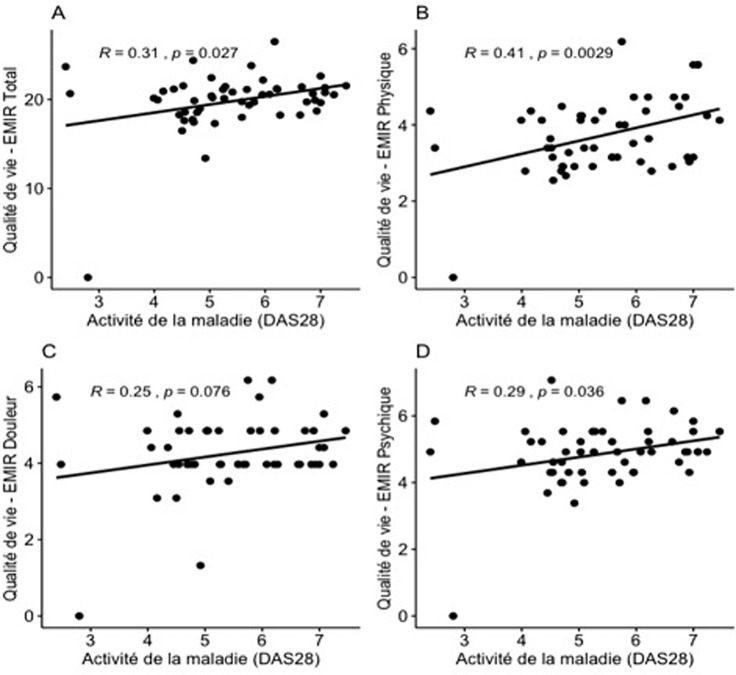
association entre l’activité de la maladie (DAS28) et la qualité de vie (EMIR); A) EMIR total, B) EMIR physique; C) EMIR Douleur; D) EMIR psychique; EMIR (échelle de mesure de l’impact de la polyarthrite rhumatoïde)

**Figure 2:**
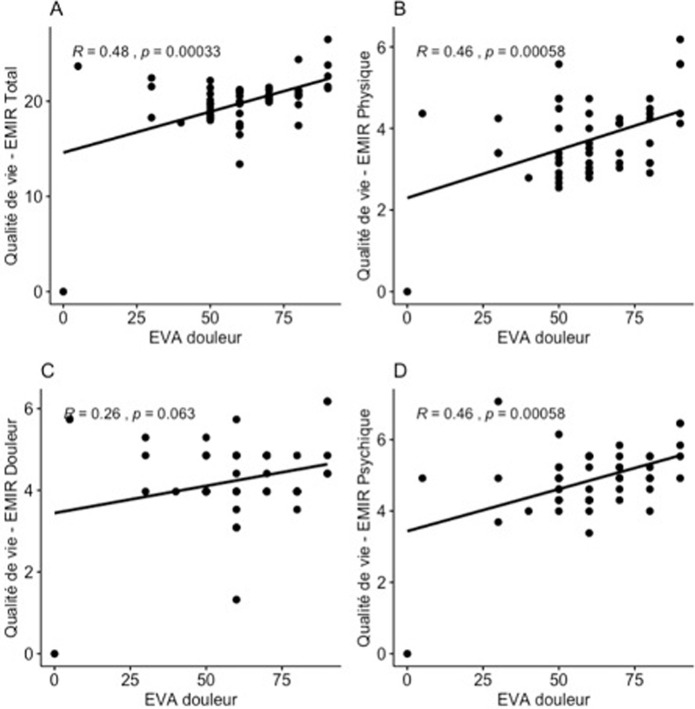
association entre l’échelle visuelle analogique-douleur (EVA-douleur) et la qualité de vie (EMIR); A) EMIR total; B) EMIR physique; C) EMIR douleur; D) EMIR psychique

Le score EMIR total était de 5,06±0,50 considéré comme une qualité de vie relativement altérée. L´impact de la maladie était plus marqué sur le composant psychique (6,78±0,99) et sur le composant douleur (5,37±0,99). Le composant travail était le moins affecté (4,03±0,98) (Tableau 2). Nous avons retrouvé un lien significatif entre le DAS28 et le score EMIR (EMIR Total: p=0,027; R=0,31; EMIR Physique: p=0,0029; R=0,41; EMIR Douleur: p=0,076; P=0,25; EMIR Psychique: p=0,036; R=0,29) (Figure 1). De même il y avait un lien significatif entre l´EVA-douleur et le score EMIR (EMIR Total: p=0,00033; R=0,48; EMIR Physique: p=0,0058; R=0,46; EMIR Douleur: p=0,063; R=0,26; EMIR Psychique: p=0,00058; R=0,46) (Figure 2). On n´a pas mis en évidence de lien significatif entre l´EVA-douleur et l´EMIR social. On n´a pas retrouvé de lien significatif entre le score EMIR et les autres paramètres de la maladie.

**Tableau 2:** évaluation de la qualité de vie des patients atteints guinéens et camerounais atteints de PR

Variables	Guinée (n=42)	Cameroun (n=10)	Global (n=52)
**Reclassement professionnel (oui)**	8/24(33,33%)	2/6 (33,33%)	10/30(33,33%)
**Score de Steinbrocker**			
Classe I	1(2,38%)	0(0%)	1(1,92%)
Classe II	6(14,29%)	5(50%)	11(21,15%)
Classe III	29(69,05%)	4(40%)	33(63,46%)
Classe IV	6(14,29%)	1(10%)	7(13,46%)
**Score EMIR**			
EMIR physique	4,40±1,06	4,52±0,66	4,43±0,99
EMIR douleur	5,54±0,82	4,64±1,34	5,37±0,99
EMIR psychique	6,85±0,99	6,47±0,99	6,78±0,99
EMIR social	4,61±0,95	5,02±1,14	4,69±0,99
EMIR travail	4,03±0,94	4,04±1,18	4,03±0,98
EMIR total	5,08±0,45	4,94±0,69	5,06±0,50

**EMIR:** échelle de mesure de l´impact de la polyarthrite rhumatoïde, **Global:** ensemble des patients. Les données sont représentées par la moyenne (± écart-type) pour les variables quantitatives et l´effectif (fréquence) pour les variables qualitatives.

## Discussion

Les caractéristiques de la PR retrouvées dans cette étude rejoignaient la littérature: la prédominance féminine, l´âge jeune de début de la maladie, la prédominance des formes séropositives au facteur rhumatoïde [15,16]. Le délai diagnostique long était lié au retard à la consultation. Dans ces pays, les patients consultent souvent les tradipraticiens avant les médecins. Ainsi, la majorité des patients sont diagnostiqués avec une PR de forte activité [4,9]. Cette forte activité de la maladie a un impact sur les capacités fonctionnelles des patients et sur leur qualité de vie. Il y avait une répercussion sur les conditions financières des patients ainsi que sur les conditions socio-professionnelles. L´impact financier et sur le travail de la PR a aussi été retrouvé en France et au Maroc [17-19]. En l´absence de système de couverture sociale dans notre contexte, l´arrêt du travail équivaut à la perte de tout revenu. Les patients sont alors pris en charge par la solidarité familiale. Il faut tout de même noter que le pourcentage des patients ayant des répercussions socioprofessionnelles liées à l´activité de la maladie est retrouvé dans une série française, 34% des patients avaient bénéficié d´une retraite anticipée [18]. Malgré le handicap fonctionnel, la PR impactait moins le travail que les autres composants (physique, douleur et psychique). Le composant social était moins affecté aussi. Cela pourrait s´expliquer par le fait que les malades ne perdent pas le lien avec leur entourage familial. L´impact de la maladie était plus marqué sur le composant psychique et le composant douleur. Une prévalence importante de la dépression au cours de la PR (45%) a été rapportée [20,21]. Une des études avait retrouvé une forte corrélation du score DAS 28 avec la dépression [20] comme chez nos patients où il y avait une corrélation entre DAS 28 et l´EMIR psychique. Le DAS28 était aussi lié de façon significative aux composants douleur, physique et à la qualité de vie globale. Une étude taiwanaise avait retrouvé que ce score composite affectait tous les domaines de la qualité de vie [22].

## Conclusion

La PR affecte la qualité de vie des patients en Guinée et au Cameroun notamment sur le plan psychique. Cet impact était lié à la douleur (EVA-douleur) et à l´activité de la PR (DAS 28), d´où la nécessité d´un bon contrôle de la maladie. Les résultats de cette étude pilote devront être confirmés par une étude d´une plus grande ampleur.

### Etat des connaissances sur le sujet

- La PR est parmi les pathologies qui ont suscité beaucoup d´intérêt dans l´évaluation (en Afrique, le questionnaire EMIR a seulement été utilisé au Maghreb);
- La PR altère la qualité de vie des patients;
- L´altération de la qualité de vie est liée à l´activité de la maladie.

### Contribution de notre étude à la connaissance

- Cette étude confirme le lien entre la sévérité de la maladie et l´impact sur la qualité de vie des populations subsahariennes affectées. La faiblesse des systèmes de sécurité sociale en Afrique amène les proches des patients de leur venir en aide en cas de perte du travail;
- Nous rapportons le lien entre des domaines précis de la qualité de vie chez des patients subsahariens (étude multicentrique) et l´activité de la polyarthrite rhumatoïde d´une part ; entre la qualité de vie et l´EVA-douleur d´autre part.
